# Implementing a knowledge management system for public health emergencies: insights from the WHO European Region COVID-19 response

**DOI:** 10.3389/fpubh.2025.1721626

**Published:** 2026-01-12

**Authors:** Miranda Tran Ngoc, Daphna Raz, Mark Shapiro, Mohamed Abdalla Elamein Boshara, Catherine Smallwood

**Affiliations:** WHO Regional Office for Europe, Copenhagen, Denmark

**Keywords:** knowledge management system (KMS), public health emergencies, COVID-19, public health emergency management (PHEM), health security

## 1 Introduction

### 1.1 Knowledge management as a pillar of public health emergency management

Public Health Emergency Management (PHEM) is the developing field of practice that utilizes specific sets of knowledge, techniques, and organizing principles necessary for the effective management of complex health events (1). Though public health emergencies are not new, they are becoming more frequent and severe (2). In the field of PHEM, getting the right information and analytics, to the right people, in an appropriate format, within a given timeframe to support decisions, can improve health as well as economic and societal outcomes (3). Knowledge Management (KM), and more specifically, Knowledge Management Systems (KMS) facilitate these processes by capturing, organizing, and retrieving knowledge assets through technological platforms and organizational processes (4). In addition, they play a key role in strengthening organizational learning (5).

### 1.2 KM frameworks and their limitations in PHEM

While KM frameworks like the SECI Model (6), Wiig's KM Framework (7), Bukowitz and Williams' KM Process Framework (8), Meyer and Zack's KM Cycle (9), and Boisot's I-Space KM Model (10) offer valuable insights, they may face limitations in public health emergency management. These frameworks may not fully account for the rapid changes and urgent decision-making required in emergencies, and the large-scale coordination and dynamic nature of public health crises can further challenge their scalability and adaptability. Limited resources during emergencies can make implementing comprehensive KM frameworks difficult, and many may not consider the specific cultural, social, and political factors influencing knowledge sharing. Moreover, these frameworks are often designed for routine operations and may not fully address the needs of emergency management.

The COVID-19 pandemic has underscored the significance of KM in managing widespread health crises (11–15). However, gaps remain, including a lack of real-world case studies, limited global mechanisms for cross-border knowledge sharing, and the need for distinct KM approaches for natural disasters vs. infectious disease outbreaks (3, 5, 12, 13, 16–18). Additionally, there is a scarcity of research linking KM to health emergencies arising from armed conflicts or other complex scenarios (5, 16). Addressing these gaps through further research and documentation is essential for guiding evidence-based practices in public health emergencies (5).

This article provides an opinion-based analysis of KMS implementation during the COVID-19 pandemic in the WHO Regional Office for Europe and identifies lessons for strengthening KM in PHEM.

## 2 Implementing KM in emergencies: the WHO/Europe COVID-19 case

The WHO Regional Office for Europe's response to COVID-19 provides a practical example of applying KM principles in real-time to address large-scale public health challenges. Activating the incident management system (IMS) for SARS-CoV-2 on January 23, 2020, WHO used a standardized approach to manage the emergency response, which is scalable, flexible, and interoperable across the organization. Initially lacking an emergency response KMS, a small team was setup to support the regional Incident Manager (IM) with information management, planning, and reporting. This team quickly developed the system and tool needed to manage the surge of information starting in March 2020.

The WHO Regional Office for Europe KMS was designed and implemented to enhance information sharing, collaboration, maintain situational awareness with the incident management team, document WHO/Europe's COVID-19 operations and lessons learned, and share evidence-based guidance to country operations.

Within PHEM, Public Health Emergency Operation Centers (PHEOCs) or Emergency Operations Centers (EOC) are crucial for managing information, enabling multiagency coordination, and supporting emergency response activities (16). This KMS initiative became the backbone of WHO's European Regional EOC platform, and the foundation of the EOC systems and products that are currently used across multiple emergencies by WHO European Region or WHO Regional Office for Europe. Developing a KMS during a health emergency demands agility, collaboration, user-centered design and leadership buy-in.

At WHO European Region, the implementation team built the KMS in real-time based on operational and strategic response needs. The design and functionality were identified through regular informal focus group discussions with the senior response leadership team, including the Regional Emergency Director and Regional IM. WHO's IMS structure, encompassing nine technical areas, was fully involved in the KMS focus groups. This iterative, inclusive approach fostered ownership and ensured the KMS remained relevant and responsive - offering a model for future emergency responses.

## 3 Knowledge management processes in PHEM for WHO/Europe

Initially, improvised tools such as Microsoft (MS) Teams, email inboxes, and SharePoint trackers were used. Over time, the KMS evolved to incorporate more structured tools to support PHEM. The development process for these tools is described in the following sections.

### 3.1 Knowledge acquisition

WHO/Europe's KMS increasingly relies on technology and digitization - a shift accelerated by COVID-19 restrictions on physical interactions. The early pandemic response exposed significant gaps in existing systems, highlighting the need for a comprehensive redesign. WHO formed remote or hybrid teams, utilizing MS Teams and SharePoint for enhanced collaboration and information accessibility. This transition improved work processes, communication, and created a favorable environment for the development of a KMS to help gather information from various sources in a singular location. WHO leveraged these tools to facilitate remote collaboration and acquire data or information (19).

During the peak of the COVID-19 response, information was vast and rapidly evolving. Critical information on the epidemiological situation, operations, and support requests were dispersed across multiple locations. With this increase in activity, a dedicated shared email inbox centralized all COVID-19 correspondence. This inbox used a color-coding system to indicate actions taken, enhancing efficiency in information management and timely task distribution. These measures helped to capture critical operational information and establish situational awareness.

To capture additional information on ongoing response activities, Knowledge Management Focal Points (KMFPs) were appointed to each IMS technical area to collect data and information to better support response documentation.

### 3.2 Knowledge refinement

As the emergency intensified, the number of technical support operations and deployments across the region increased. Initially, reporting for these activities was compiled through a PowerPoint template, with technical areas providing weekly updates.

However, the data collection system evolved to include various tools and standardized formats, transitioning from PowerPoint to a Microsoft Word reporting form with advanced features such as drop-down lists, calendars, and macros. These forms captured granular operational data, including deployments and challenges, and later expanded to collect detailed information on activities, major achievements, and publications. The KMFPs, and WHO Country Office focal points were the main users involved in the systematized data collection process reporting weekly on the COVID-19 situation and response activities.

In 2022, multiple emergencies emerged which impacted the WHO European Region simultaneously. To meet these emergency response needs, a versatile electronic reporting tool was developed, designed to be suitable for all-hazard scenarios including disease outbreaks, natural disasters, and population movements.

*ActivityInfo* (19) software was adopted to further advance data management with automated data quality features, built-in validation, and custom data quality rules. Establishing data quality check systems, both manual and automated were critical in refining the acquired data. The software's audit trail features logged changes and tracked data quality over time, supporting data compliance and monitoring. The software was chosen for country-level reporting and integrated with MS tools at the regional level, replacing previously existing forms. Different health emergencies have varying data collection and reporting needs, based on response plans and donor funding. An assessment of the *ActivityInfo* electronic multi-emergency reporting tool identified several advantages over MS Word and Excel macros previously used, including centralized data storage, improved data validation, enhanced collaboration, advanced reporting capabilities, robust security, and better data standardization.

Throughout the KMS, the implementation of robust data governance processes ensured high quality and consistent data. This included establishing a shared understanding of the KMS through standardized definitions and SOPs, reviewing classification codes for accuracy, delineating KMFPs roles and responsibilities, deconflicting data reports, and defining data standards.

Applying data mining strategies, particularly the application of classification codes for response activities was part of the knowledge refinement process. This involved developing a common set of metrics to code all response activities, identified through focus group discussions and internal analysis of ongoing support. These classification codes were applied by KMFPs allowing for consistent data collection and capturing of operations across different technical areas. These measures were instrumental in the systemization and development of the KMS, guaranteeing data quality, reliability, and usability.

### 3.3 Knowledge storage and retrieval

The KMS enhanced systematic knowledge documentation across the organization by storing all documents in a standardized set of folders. This approach facilitated easy knowledge retrieval and advanced resource searches. Implementing a standardized document filing system was a crucial part of the KMS, and its application across multiple emergencies ensured predictability and consistency in knowledge retrieval. Mapping information flows also helped to streamline the integration and retrieval of knowledge from various sources including emergency reporting tools and the shared inbox, which were then reflected in knowledge-sharing products.

The collected data was merged and saved in a data warehouse, allowing for efficient storage and management of refined knowledge received through *ActivityInfo* and other reporting forms. This setup enabled the development of dashboards that illustrated and quantified WHO support to Member States.

Business intelligence tools played a key role in understanding organizational performance during the COVID-19 response by providing a quantitative overview of operations and data visualization. Creating dashboards to showcase WHO support to Member States required extensive consultations to determine visualization and design needs. The final dashboard retrieved data from various response areas, enabling self-service reporting and in-depth analysis (20). This enhanced real-time reporting, decision-making, and overall organizational performance.

### 3.4 Knowledge distribution

The shared inbox proved successful in triaging and managing information. The next step was to create a dynamic knowledge-sharing tool. Initially, a MS Teams channel served as a virtual support team, providing daily updates, addressing FAQs, and sharing resources with WHO offices in the Region. Launched at the pandemic's start, this channel evolved into tools such as a daily newsletter sent from the shared inbox.

This “Daily Digest” newsletter curated critical new resources, including guidance documents, epidemiological data, talking points, and other essential information for WHO Country Offices and response teams. This reliable resource mitigated information overload amid the daily influx of new research, data, and misinformation.

In early 2021, weekly KMFP meetings were initiated to enhance knowledge distribution processes. These meetings fostered an internal community of practice, promoting trust, inclusiveness, and collaboration among participants. They addressed various aspects of knowledge sharing, including data quality issues and potential improvements, and facilitated the dissemination of experiences, lessons learned, and innovative ideas. Governance processes were formalized with co-designed terms of reference, standard meeting agendas, and regular action points, ensuring the KMFP group remained focused and effective in distributing knowledge across the organization.

### 3.5 Knowledge presentation and use

The KMS facilitated the consolidation and presentation of information that demonstrated how the WHO Regional Office for Europe supported Member States during the COVID-19 pandemic and other emergencies. Using standardized templates and consistent branding, particularly for COVID-19-related products, the system promoted clear, recognizable knowledge sharing, contributing to greater transparency and trust.

This structure supported real-time decision-making and provided leadership with a comprehensive understanding of organizational performance. It established a common operational picture that enhanced the effectiveness of emergency response efforts. Although primarily designed for internal WHO leadership, the KMS was also accessible and utilized by personnel at both Regional and country levels.

The implementation of the KMS standardized documentation processes and improved situational awareness for leadership. It informed key outputs such as response timelines (21), regional reports (22), donor reports, and operational reviews. These outputs drew upon knowledge stored in the data warehouse, documenting response activities and facilitating knowledge exchange both within WHO and with external stakeholders.

Furthermore, the system improved the efficiency of information flow throughout the organization. It reduced reporting burdens, expedited information retrieval, ensured timely data provision, and effectively visualized WHO Regional Office for Europe's emergency response. By providing a centralized platform for data storage and knowledge sharing, the KMS strengthened the documentation of health emergency responses at both country and Regional levels.

### 3.6 Evaluation and continuous improvement

The effectiveness of the KMS can be evaluated through key metrics, including user satisfaction, knowledge utilization, and continuous improvement (23). This demonstrates that the KMS was well-integrated within the organization, aligned with organizational needs and effectively supports PHEM. The COVID-19 response led to significant organizational changes at WHO, empowering the emergency operations team to expand these systems. Knowledge utilization metrics showed that the COVID-19 SharePoint was visited by response team members 448,499 times, with 867 unique views, indicating high usage rates and integration into daily workflows.

The COVID-19 emergency fostered team resilience, motivation, innovation, and adaptability within the KM team. The implementation team had a low turnover rate compared to the KMFPs, preventing disruptions in development. The core team consistently operated through various emergencies, gaining experience, refining processes, and collaborating closely from 2020 to 2024.

Regular KMFP meetings fostered an inclusive environment for co-created knowledge, focusing on the end user. These forums leveraged internal knowledge from individuals with diverse expertise, promoting ownership and integrating the process within the organization. A user satisfaction survey conducted in September 2023, following the conclusion of the acute phase of the COVID-19 response, indicated high levels of comfort with the digital reporting tools among all respondents, several years after their initial rollout. Furthermore, 80% of participants reported feeling adequately trained, well-supported by the KM implementation team, and clear about their responsibilities within the KMFP terms of reference.

**Figure 1:**
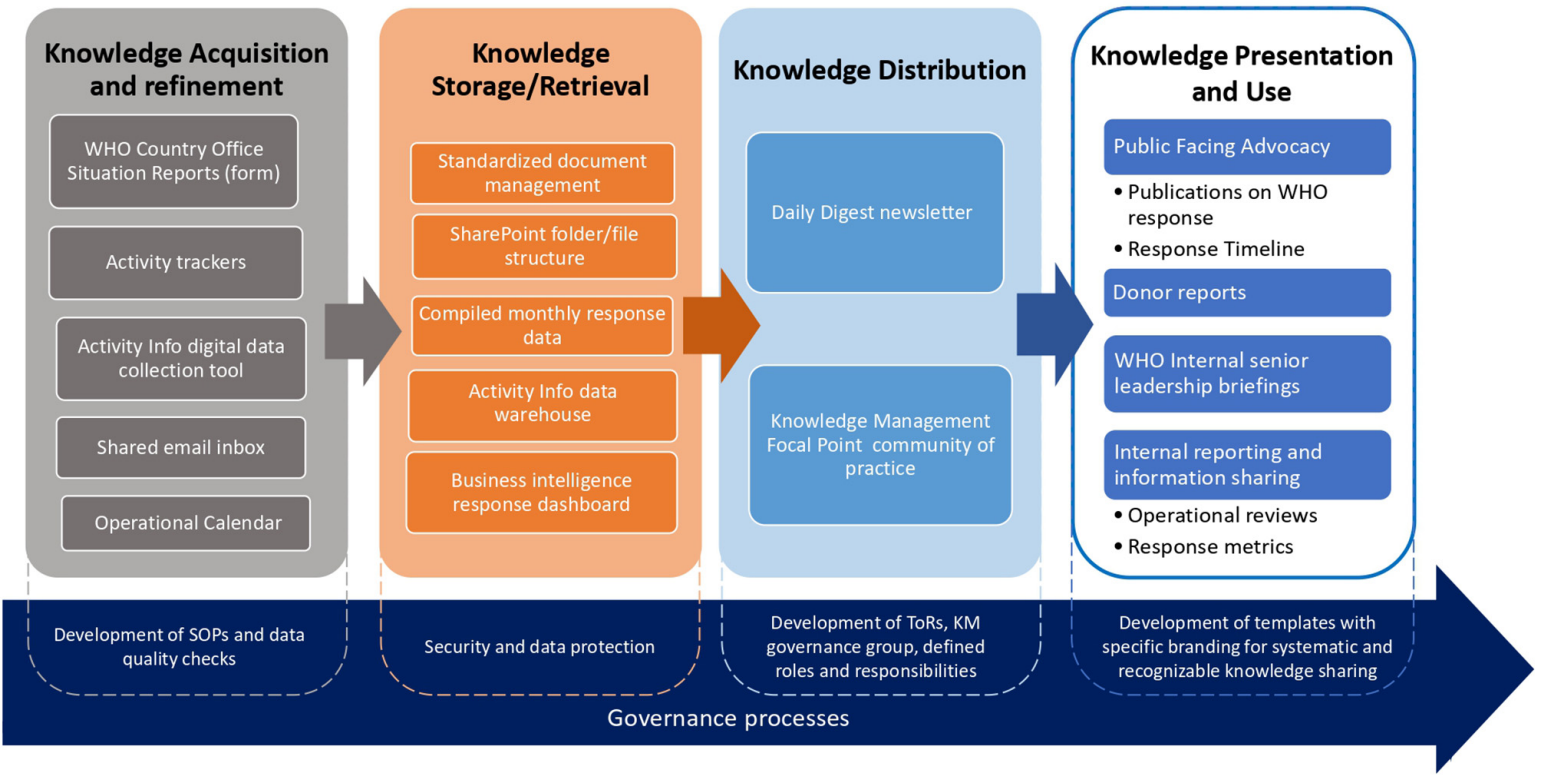
WHO COVID-19 KMS mapped across the five components of the KM cycle. Source: Adapted from (9).

Although most of the KMS was designed and implemented while actively responding to emergencies across the WHO European Region (Figure 1) it was ultimately aligned with the KMS ISO requirements (17). The iterative development of the KMS prioritized user needs, allowing for continuous improvement through regular monitoring and adjustments based on feedback and performance data. This approach maintained alignment with organizational objectives.

High risk tolerance within the team and endorsement by WHO leadership enabled innovation, which in turn supported regular testing and adaptation via prototypes, collaborative ideation, mentoring, and agile methods to meet various informational needs. This culture of continuous improvement is exemplified by the number of response dashboard designs and the quality of the reporting products that were implemented and improved on between 2020 and 2024.

In responding to the war Ukraine, the KMS was stress tested and the differing nature of activities and emergencies (armed conflict vs. infectious disease) revealed new areas for design and development of the KMS. This process was another learning opportunity as a separate emergency response team was established for WHO's response to the war in Ukraine, which created a parallel KMS within the Regional office. Having two separate KMS fragmented the developing KM culture, which created additional challenges, but also provided valuable lessons for strengthening the system amid multiple public health crises. The development and implementation of a single online tool for multi-emergency data collection significantly improved the KMS ability to simultaneously address emergencies of all -hazard.

## 4 Practical lessons learned from the WHO European Region KMS implementation

The WHO Regional Office for Europe learned several valuable lessons (Table 1) throughout the COVID-19 pandemic regarding the development and implementation of a single multi-emergency reporting system. These lessons underscore the importance of standardization, consistency, adaptability, and collaboration in managing complex global health emergencies (Table 1).

**Table 1:** Lessons identified through the WHO European Region KMS implementation.

**Number**	**Lesson identified**	**Description**
1	Implement digital systems early and ensure proficiency	Ensure that user friendly digital tools are not only established but also consistently utilized, with response teams proficient in using a fundamental set of tools (33). The foundation of the system design is based on the existing software platform and ensures easy integration and reduces the use of new tools. This centralizes information in one place and improves uptake of the KMS.
2	Standardized multi-emergency reporting and data governance	Develop online multi-emergency data collection forms to standardize information gathering, implement data governance processes and ensure high-quality data. This can streamline reporting across different types of emergencies.
3	Apply classification codes to enable cross-emergency indicators	Apply standardized classification codes for all hazards, to enable cross-cutting indicators and visualizations that use data from various emergency types. Carefully consider the addition of new classification codes before inclusion can improve visualization quality (34).
4	Build a core implementation team	Build a team that can lead the design and implementation throughout the lifecycle of the KMS development. Reducing turnover promotes trust, collaboration and psychological safety within the team, helping the team to focus on the tasks at hand and overcome implementation challenges together.
5	Advocate for strong leadership in championing the system	Strong leadership, with an appetite for innovation is essential for receiving buy-in, user feedback and design input from all stakeholders (35). The leader's strong understanding of the process, functionality and uses of KMS helped to empower the design team and ensure the necessary engagement within the response team.
6	Develop standardized processes	Develop and implement SOPs, define roles and responsibilities, and conduct regular training sessions to ensure strong governance processes and support for tool deployment. This ensures consistency in reporting practices and enhances overall KMS efficiency.
7	Build a co-designed community of practice to foster ownership	Establish a co-designed community of practice to implement the KMS on a larger scale. This approach fosters knowledge sharing, collaboration, and supports knowledge translation among users, ensuring that the community meets members' expectations and facilitates practical applications to address organizational challenges (36). Co-design supports culture change and the embedding of the KMS into the teams' workflows.
8	Establish unified channels to reduce fragmentation and overload	Implement a single, consistent channel for disseminating emergency-related information and knowledge to minimize search time and mitigate information overload.

## 5 Discussion

### 5.1 Added Value of KM in a Public Health Emergency Operations Centre

WHO's Emergency Response Framework (24) and its IMS specifically relies on the functionality of an emergency operations center, where the PHEOC is a central location for the coordination of information and resources to support incident management activities, across a variety of event types (16).

The extent of the Regional EOC in early 2020 (prior to COVID-19) was a meeting room, with limited teleconferencing functionality and no dedicated EOC staff, KMS or standardized procedures in place. The EOC's watch and alert mode was covered by the health information team, which was separate from the operational response capacity, and predominantly driven by the International Health Regulations (IHR 2005), health information and signal detection requirements (25).

The key components that make a PHEOC functional, as documented in the WHO PHEOC framework (26) and handbooks, are plans and procedures, physical infrastructure, information communication technology (ICT) infrastructure, information systems and data, as well as human resources (27). The PHEOC framework defines three types of information required for decision-making. These are incident-specific information, event information, and contextual information (26).

As the COVID-19 emerged, so did the need to support the IM with appropriate clear KM processes, procedures, tools, and information products. In building up the Region's IMS capacity, a small group was pulled together to support the regional incident manager. This “EOC team” established and maintained a highly functional regional platform, with the KM systems and tools needed to provide key operational information to the regional response team and senior WHO leadership.

Although WHO is a knowledge-driven Organization, exemplified by the 2005 publication of both a comprehensive organization-wide KM operational plan (28) and KM strategy (29), WHO had not implemented the concepts of KM for emergency response within the WHO European Region Health Emergencies Programme, and particularly not to the scale needed during the COVID-19 response. In some respects, the lack of the EOC and KMS infrastructure reflected the low number of emergencies requiring a WHO operational response prior to 2020.

The WHO European Region leadership team, made significant investment in their response capabilities, demonstrated by the creation of fixed KM and EOC roles, the upgrading of the physical EOC space and through the continuous support for this area of work. The WHO European Region EOC now functions as an established platform for the Health Emergency Programme. It is set up as a hybrid EOC, meaning it can function for both remote users and for the team based in the dedicated EOC space. All EOC systems are cloud-based, and information is handled according to WHO ICT standards for security and information management.

The development and digitalization of the KMS, done during COVID-19 and refined in subsequent emergencies, has helped to establish an all-hazard Regional EOC, that functions during all phases of the emergency management cycle.

### 5.2 Use of artificial intelligence in knowledge management

The recent leaps in both online communication tools and artificial intelligence (AI) provide new opportunities that were not available at the time of building the initial KMS (30). The integration of AI into KMS presents transformative opportunities for PHEM. AI facilitates the development of tailored tools that can be seamlessly embedded into existing work cultures, leveraging commonly used platforms to reduce barriers to adoption (31). By automating routine and manual tasks, AI reduces resistance among users and enhances operational efficiency. It can also accelerate data governance processes, ensuring higher data quality and consistency, which is critical in emergency contexts.

Moreover, AI can support the design and implementation of KMS, particularly in settings with limited capacity or technical expertise, thereby broadening access and usability. Recent empirical evidence shows that AI-empowered KM processes significantly improve decision-making and accessibility across diverse organizational contexts (31).

AI agents can further enhance operational KM by enabling rapid retrieval and contextualization of knowledge, supporting informed decision-making during crises (32). As an example, the use of AI agents within SharePoint libraries, like those used at the WHO Regional Office for Europe, are a powerful application for enhancing knowledge retrieval. By applying these agents, users can efficiently search and access relevant information from vast document repositories and data warehouses. By interpreting the users prompts, AI agents can provide precise, context-aware responses based on the structured and unstructured knowledge stored. At WHO, this helps to quickly retrieve information during emergencies—supporting faster decision-making.

Collectively, these capabilities position AI as a catalyst for more agile, inclusive, and effective KMS in public health emergencies. However, the application of AI tools in public health emergencies requires careful attention to data privacy and protection. This is particularly critical due to the highly sensitive and often confidential nature of information exchanged during meetings, through email communications, and within official documents. Such data may include personal health details, operational strategies, or other protected information that, if mishandled, could lead to serious ethical, legal, or reputational consequences.

### 5.3 Limitations to KMS application

Throughout the KMS building process, KM was often confused with health information management or information management. This confusion highlights the need for cultural change—recognizing KM as a people-centered approach that integrates process and technology to foster knowledge sharing and use.

The KMS was developed in real time during an emergency, without fully established monitoring and evaluation systems. As a result, data to assess its efficiency and impact remain limited.

The system relies on data input across the response teams, including country office incident management teams. Reporting fatigue by teams and technical staff, along with staff (user) turnover was a challenge that required monthly reminders, regular trainings and the integration of the KMS into all response team workflows.

Additionally, the unique organizational culture of the WHO European Region Emergency Programme, which enables ideas to flow from any level of the organization to senior management, coupled with appropriate risk appetite, helps to drive innovation. This context may limit the broader applicability of this approach to other organization's contexts. The systems, set-up, and processes outlined are experiences of the WHO Regional Office for Europe and would need adaptation if implemented elsewhere.

Moreover, it is worth noting that the WHO KMS is not a finished product but remains dynamic and under continuous enhancements, which would likely be the case for most emergency related KMS.

## 6 Conclusions and recommendations

What began as an organic and improvised solution, has evolved into a foundational system embedded within the WHO European Regional EOC. While every organization is different, our experience demonstrated that a strong KMS can make a huge difference, especially for emergency teams that require high levels of coordination, collaboration, and information sharing. The KMS provides predictable, reliable, scalable and appropriate tools to support all levels of the incident management team.

While emergencies do provide opportunities to leapfrog technology and implement innovation, having the governance (e.g., WHO Emergency Response Framework) and processes in place are as important as implementing new technology solutions.

The recommendations from the WHO European Region experience include establishing a KM strategy and system before an emergency, utilizing it from the beginning of any response, and committing to continuous improvement. This requires the implementation of governance processes that ensure the system is part of regular workflows and remains fit for purpose. Equally important for the sustainability of a KMS, is building a KM culture rooted in a community of practice, supported by leadership and sufficient resources

Knowledge management systems should be established as a key pillar of one's emergency system, especially when considering a One Health response that requires multi-sector coordination and information sharing. Having a KMS can be a good proxy indicator for emergency management system readiness and can be connected to a wider investment in digitalization and emergency response information management systems.

Continuing developments in AI are creating new opportunities, tools and applications that need to be reviewed and if appropriate, designed and integrated into the KMS. As KM for PHEM expands, consistent use in emergency contexts and systematic documentation of lessons learned will be essential. Capturing these insights and applying innovative tools will help ensure more adaptive, evidence-based, and resilient public health emergency management.

As a growing field of work, additional research is needed in the application of KMS for PHEM. Gaps remain, including a lack of real-world case studies and scarcity of research linking KM to health emergencies arising from armed conflicts or other complex scenarios (5, 16). Addressing these gaps through further research and documentation is essential for guiding evidence-based practices in public health emergencies.

The WHO European Region response team has benefitted by having a KMS, which directly improved the speed and predictability of WHO's Regional responses. The aim of this paper is to strengthen national emergency response systems and support PHEOC teams. The WHO Regional Office for Europe will continue to advocate for and explore the application of KMS to enhance emergency coordination and response.
